# Effects of an intensive lifestyle intervention and the role of sleep in people living with HIV and prediabetes: a pilot and feasibility study

**DOI:** 10.1186/s13104-021-05558-z

**Published:** 2021-04-17

**Authors:** Hataikarn Nimitphong, Somnuek Sungkanuparph, Chatvara Areevut, Sunee Saetung, Ratanaporn Jerawatana, Amornrat Hathaidechadusadee, Supaporn Somwang, Wanabhorn Tongchom, Nampeth Saibuathong, Jandanee Sakmanarit, Orawan Pichitchaipitak, Angsana Phuphuakrat, Sirimon Reutrakul

**Affiliations:** 1 Department of Medicine, Faculty of Medicine Ramathibodi Hospital, Mahidol University, Bangkok, Thailand; 2 Chakri Naruebodindra Medical Institute, Faculty of Medicine Ramathibodi Hospital, Samut Prakan, Thailand; 3 Nursing Department, Faculty of Medicine Ramathibodi Hospital, Mahidol University, Bangkok, Thailand; 4 Division of Nutrition and Dietetics, Faculty of Medicine Ramathibodi Hospital, Mahidol University, Bangkok, Thailand; 5 Nutrition Science Group, Faculty of Medicine Ramathibodi Hospital, Mahidol University, Bangkok, Thailand; 6 Division of Endocrinology, Diabetes and Metabolism, University of Illinois At Chicago, Chicago, IL USA; 7 Division of Endocrinology and Metabolism, Department of Medicine, Faculty of Medicine Ramathibodi Hospital, Mahidol University, Rama VI Road, Ratchathewi, Bangkok, 10400 Thailand

**Keywords:** HIV, Intensive lifestyle intervention, Prediabetes, Sleep

## Abstract

**Objectives:**

Prediabetes is prevalent in people living with HIV (PLWH). Insufficient and irregular sleep are linked to abnormal glucose metabolism. This study aimed to investigate the differences in sleep characteristics between PLWH with and without prediabetes, determine the acceptability/feasibility and effects of a pilot six-month intensive lifestyle intervention (ILI) program on glucose metabolism in those with prediabetes, and determine how sleep modulates these effects.

**Results:**

Thirty-nine PLWH (20 normoglycemia and 19 prediabetes) participated. There were no differences in sleep characteristics between individuals with normoglycemia and prediabetes. Next, thirteen individuals with prediabetes completed a six-month ILI program. The ILI program resulted in significant body weight reduction at 6 months (63.5 ± 13.9 to 61.9 ± 14.0 kg, *p* = 0.012), which was maintained at 12 months (*p* < 0.001). Waist circumferences were significantly decreased at 12 months (85.4 ± 11.7 to 82.9 ± 12.7 cm, *p* = 0.014). An increase in sleep variability was significantly associated with an increase in 2-h plasma glucose, independent of changes in BMI (b = 0.603), and physical activity (b = 0.774). This pilot study suggested that ILI in PLWH with prediabetes is feasible and effective in improving metabolic control, with its effects possibly modulated by sleep variability. These findings should be confirmed in a larger study to reduce diabetes risk in this population.

**Trail registration:** ClinicalTrial.gov, NCT03545217 (date of registration: May 22, 2018)

## Introduction

Type 2 diabetes mellitus (T2DM) is prevalent in people living with HIV (PLWH) [1, 2]. In Thailand, the prevalence of T2DM and prediabetes among PLWH are 14% [3] and 27.5% [4], respectively. The prediabetes prevalence in this group was higher than the 10.6% rate in the general Thai population [5].

Intensive lifestyle interventions (ILI) are effective in reducing diabetes rates and are generally recommended to the general population and PLWH with prediabetes [6–8]. Whether or not the unique characteristics of PLWH affects the outcomes is unknown. To date, there have been only a few studies that explored the effectiveness of ILI in PLWH with impaired glucose tolerance. A non-randomized study of 28 PLWH that had impaired fasting glucose showed that ILI for 6 months improved glucose, blood pressure and lipids levels [9]. Another study showed that a supplementation of pioglitazone to an exercise program for 4 months improved insulin sensitivity in PLWH that had insulin resistance and central obesity [10]. Therefore, understanding the acceptability and efficacy of ILI along with factors that may modulate the responses, is crucial in reducing the diabetes risk in this group.

In people with normoglycemia, prediabetes and T2DM, sleep disturbances, including insufficient sleep, poor sleep quality and increased sleep variability (varying day-to-day sleep timing or duration), have been linked to abnormal glucose metabolism [11] and cardiometabolic risks [12–14]. Studies in non-HIV people have shown that prediabetes individuals had shorter sleep durations (< 5–6 hours (h)/night) [15, 16] when compared to normoglycemic individuals. When compared to non-HIV infected people, PLWH had a higher prevalence of poor sleep quality [17, 18]. However, there has been no study exploring the differences in sleep characteristics between PLWH with normoglycemia vs. prediabetes. ILI could potentially improve sleep. For example, exercise has been shown to improve sleep quality in patients with insomnia [19] and fibromyalgia [20]. The potential mechanisms include thermoregulation, body restoration, energy conservation hypotheses [21], and regulation of pro-inflammatory cytokines [22] in response to exercise. In addition, weight loss could improve obstructive sleep apnea severity which could improve sleep quality and duration [23]. There has only been limited studies on whether or not sleep characteristics modulate the response of diabetes prevention interventions.

The objectives of this study were to investigate the differences in sleep characteristics between PLWH with and without prediabetes. Furthermore, we tested the feasibility and effects of a pilot six-month ILI program on glucose metabolism in those with prediabetes and explored whether sleep characteristics modulated the response to ILI. The acceptability of the program was evaluated. The results of this study will provide information to design future larger studies aimed at reducing diabetes risks in PLWH with prediabetes.

## Main text

### Materials and methods

This study was conducted among PLWH who received antiretroviral therapy (ART) and were being followed at an infectious disease clinic in a university hospital in Bangkok, Thailand. Thirty-nine PLWH (20 normoglycemia and 19 prediabetes) from a previous cross-sectional study for the prevalence of prediabetes among PLWH [24], who were willing to participate, were included in this study. Prediabetes was defined as follows: hemoglobin A1c (HbA1c) ≥ 5.7- < 6.5%, fasting plasma glucose (FPG) levels < 126 mg/dL, and 2-h plasma glucose (2 h-PG) levels < 200 mg/dL following a 75-g oral glucose tolerance test (OGTT) [25]. Exclusion criteria included medical problems causing limited physical activities (for example; active coronary artery disease, obstructive lung disease) [24, 26], the use of glucose lowering medications, known obstructive sleep apnea, performing shift work and pregnancy. This study adhered to CONSORT guidelines.

#### Study protocol

This study consisted of two parts. In the first part, a cross-sectional study, 39 PLWH (20 normoglycemia and 19 prediabetes) were assessed for their medical histories and anthropometric measurements. OGTT, HbA1c, and objective sleep assessments were performed (see below).

In the second part, a pilot interventional study of a single open trial, 14 of 19 individuals with prediabetes (74%) participated in a six-month ILI program. Repeated anthropometric measurements and glucose homeostasis were performed at the end of the program and at 12 months in 13 PLWH with prediabetes who completed the ILI program. Sleep patterns were reassessed at 6 months.

#### Glucose homeostasis assessment

After an overnight fast, 75-g of glucose was given orally. Blood samples were obtained at 0 min for HbA1c, and at 0, 30, 60, 90, and 120 min for glucose and insulin. To evaluate early changes in glucose homeostasis, the markers of glucose metabolism, including the Matsuda index [27], HOMA-IR [28], the insulinogenic index [29] and the Disposition Indices [30] were calculated.

#### Objective and subjective sleep assessment

Individuals wore an Actiwatch 2 wrist activity monitor (Philips Respironics, Bend, Oregon) for 7 days. Sleep duration was defined as the amount of actual sleep obtained at night, and sleep efficiency as a percentage of time in bed spent sleeping. Mid-sleep time (MST) was defined as the midpoint between sleep start and wake time. The standard deviations (SDs) of sleep duration and MST were calculated and used as sleep variability parameters as previously described [13]. For each individual, the mean across all available nights was used.

Sleep quality in the previous month was assessed using a validated Thai version of the Pittsburgh Sleep Quality Index (PSQI) [31].

#### Physical activity assessment

The physical activity was assessed by the Global Physical Activity Questionnaire (GPAQ) version 2 and measured as Metabolic Equivalents (METs) [32, 33].

#### Quality of life

Quality of life was measured using the European Quality of Life Measure-5 Domain-5-Level (EQ-5D-5L) [34, 35] and the visual analog scale [VAS; scale ranged from 0 (the worst) to 100 (the best)].

#### The six-month intensive lifestyle intervention (ILI) program

This pilot six-month ILI program was led by a multidisciplinary team of diabetes nurses and dieticians, and included a follow up at 12 months. The program was a group-based activity adapted from the Diabetes Prevention Program (DPP)-Thailand project, which was shown to be effective in improving glycemic parameters [36]. The goals of the program were to target three main aspects of a healthy lifestyle including healthy eating (aiming 7% weight loss in overweight individuals), exercise (150 min of aerobic exercise weekly) and healthy coping (for example, practicing in spiritual and mindfulness, emotional management). This modified program consisted of five one-day sessions which met every six weeks over the 6-month period, and supplementary weekly telephone follow-ups (10–15 minutes). Each session included instructions from the health care team, through workshop activities and the setting of mutually-agreed behavioral goals. In-between-visit telephone follow-ups were aimed at encouraging goal achievement, discussion of problems and enhancing health knowledge.

To evaluate the acceptability of the intervention, questionnaires which addressed the participants’ satisfaction of their experience were administered 4 times, at the end of each session (1, 2, 3 and 4).

### Statistical analysis

Comparisons of the differences between normoglycemia and prediabetes groups were performed by Student t Test, Mann–Whitney U test and Pearson’s chi-squared test, as appropriate. Comparison of metabolic parameters at baseline and 6 months, and baseline and 12 months, were performed by paired-samples T-test or Wilcoxon Signed Ranks Test. Spearman’s correlation was used to investigate the association between changes in body mass index (BMI), physical activity, sleep parameters and changes in metabolic parameters at 6 months compared to baseline. A multiple linear regression analysis was further performed to investigate the association between changes in sleep parameters and changes in metabolic parameters after adjusting for the changes in BMI and physical activity. Analyses were performed using SPSS statistical software package, version 18.0 (SPSS, Chicago IL, USA).

## Results

### Baseline demographics, metabolic and sleep characteristics in normoglycemic and prediabetic individuals

Thirty-nine PLWH (20 normoglycemia and 19 prediabetes) with a mean age of 51.5 ± 6.0 years were included (Table 1). There were no differences in clinical characteristics between the two groups (Table 1). As expected, individuals with prediabetes had higher FPG, 2 h-PG, HbA1c, and HOMA-IR than those in the normoglycemia group. Other indices obtained from OGTT (Matsuda, Insulinogenic and Disposition Indices), lipid profiles and all sleep parameters were similar between the two groups.

**Table 1:** Baseline clinical characteristics of 20 PLWH with normoglycemia and 19 PLWH with prediabetes

Characteristics	Normoglycemia *(n* = *20)*	Prediabetes *(n* = *19)*	*P* value
**Demographic and anthropometric parameters**			
Age (years)	51.8 ± 6.6	51.2 ± 5.5	0.767
Male gender, number (%)	13(65)	12(63.2)	1.00
Family history of diabetes, number (%)	4(20)	9(47.4)	0.096
History of smoking, number (%)	10(50)	6(31.6)	0.333
History of alcohol drinking, number (%)	16(80)	11(57.9)	0.176
Underlying diseases, number (%)			
Dyslipidemia	9(45)	7(36.8	0.748
Hypertension	4(20)	3(15.8)	1.00
NAFLD	0	1(5.3)	0.487
Cancer	0	0	NA
Others^a^	0	0	NA
BW (kg)	62.7 ± 12.8	63.2 ± 12.5	0.912
BMI (kg/m^2^)	23.1 ± 4.6	24.1 ± 4.1	0.500
SBP (mmHg)	130.5 ± 15.5	130.1 ± 14.7	0.935
DBP (mmHg)	81.3 ± 8.1	82.1 ± 10.9	0.781
WC (cm)	84.8 ± 10.8	85.3 ± 11	0.901
Neck circumference (cm)	35 ± 3.1	35.0 ± 4.0	0.947
**HIV-related parameters**			
Duration of HIV infection (years)	15.2 ± 6.1	14.5 ± 5.5	0.729
Type of ART regimen, number (%) NNRTI-based PI-based Others	13(65) 7(35) 0	13(68.4) 6(31.6) 0	1.00 1.00 NA
Duration of ART (years)	11.5 ± 6.1	11.9 ± 5.3	0.843
CD4 cell counts (cells/mm^3^)	559.8 ± 264	455.7 ± 190.7	0.169
**Metabolic and other biochemical parameters**			
FPG (mg/dL)	91.8 ± 7.9	99.1 ± 14.9	0.002
2 h-PG (mg/dL)	113.5 ± 40	153.6 ± 46.9	0.007
HbA1c (%)	5.32 ± 0.21	5.99 ± 0.23	< 0.001
Matsuda Index	3.29(1.73–5.41)	2.44(1.53–3.44)	0.160^b^
HOMA-IR	2.51(1.19–3.04)	3.66(1.74–6.02)	0.044^b^
Insulinogenic Index	0.79(0.11–1.52)	0.84(0.34–1.51)	0.901^b^
Disposition Index	2.65(0.29–5.32)	1.70(0.84–3.66)	0.550^b^
Alanine transaminase (U/L)	36.6 ± 18.5	38.8 ± 21.5	0.729
Total cholesterol (mg/dL)	219 ± 42	204.3 ± 37	0.255
HDL cholesterol (mg/dL)	51.2 ± 11.6	46.8 ± 11.3	0.248
LDL cholesterol (mg/dL)	141.3 ± 36	130.6 ± 34.2	0.350
Triglycerides (mg/dL)	146.1 ± 90.8	166 ± 122.2	0.566
**Sleep parameters**			
PSQI	5(3–7)	4(3–7)	0.607^b^
Sleep efficiency (%)	81.9(78.2–85.9)	84.0(77.6–87.8)	0.627^b^
Sleep duration (min)	338.5(316.3–367.5)	354.6(332.8–388.9)	0.283^b^
SD sleep duration (min)	52.9(33.9–72.6)	51.3(34.9–76.0)	0.708^b^
SD MST^c^	0:42 ± 0:30	0:30 ± 0:18	0.123

Data was presented as mean ± SD or IQR

*PLWH* people living with HIV, *BW* body weight, *BMI* body mass index, *NAFLD* nonalcoholic fatty liver disease, *ART* antiretroviral therapy, *NNRTI* non-nucleoside reverse transcriptase inhibitor, *PI* protease inhibitor, *SBP* systolic blood pressure, *DBP* diastolic blood pressure, *WC* waist circumference, *FPG* fasting plasma glucose, *2 h-PG* 2-h plasma glucose, *HbA1c* hemoglobin A1c, *HDL* high-density lipoprotein, *LDL* low-density lipoprotein, *PSQI* Pittsburgh Sleep Quality Index, *SD* standard deviation, *MST* mid-sleep time

^a^Including cerebrovascular disease, coronary artery disease and chronic kidney disease

^b^Mann Whitney U test

^c^time presented as 24-h clock time

### Results of ILI

Thirteen PLWH with prediabetes completed the six-month ILI program and a 12-month follow-up visit. At 6 months, there was a significant reduction in BW and BMI from baseline, which was maintained at 12 months (Table 2). Waist circumferences (WC) significantly decreased at 12 months (Table 2). There were no changes in physical activity during the follow-up period. FPG non-significantly decreased at 6 months (*p* = 0.051), whereas there were no changes in 2 h-PG and HbA1c at 6 and 12 months. For OGTT-derived indices, when compared to baseline, the Matsuda index non-significantly increased at 12 months (*p* = 0.064), HOMA-IR significantly decreased at both 6 and 12 months, and an insulinogenic index non-significantly decreased at 6 months (*p* = 0.055).

**Table 2:** Compared parameters at baseline, 6 months (at the end of the program) and 12 months in 13 PLWH with prediabetes

Characteristics	baseline	6 months	*P* value (baseline and 6 months)	12 months	*P* value (baseline and 12 months)
**Demographic parameters**					
Age (years)	52.9 ± 4.8				
Male gender, number (%)	8(61.5)				
Family history of diabetes, number (%)	7(53.8)				
History of smoking, number (%)	4(30.8)				
History of alcohol drinking, number (%)	8(61.5)				
Underlying diseases, number (%)					
Dyslipidemia	4(30.5)	5(38.5)	0.317	5(38.5)	0.317
Hypertension	2(15.4)	2(15.4)	1	2(15.4)	1
NAFLD	1(7.7)	1(7.7)	1	2(15.5)	0.317
**HIV-related parameters**					
Duration of HIV infection (years)	15.3 ± 4.9				
Type of ART regimen, number (%)					
NNRTI-based	8(61.5)	8(61.5)	1	8(61.5)	1
PI-based	5(38.5)	5(38.5)	1	5(38.5)	1
Others	0	0	NA	0	NA
Duration of ART (years)	12.7 ± 5.5				
CD4 cell counts (cells/mm^3^)	405.1 ± 157.0	466.6 ± 239.3	0.146	459.8 ± 238.2	0.213
**Anthropometric parameters and physical activity**					
BW (kg)	63.5 ± 13.9	61.9 ± 14.0	0.012	62.0 ± 14.3	< 0.001
BMI (kg/m^2^)	24.3 ± 4.3	23.6 ± 4.3	0.014	23.7 ± 4.4	< 0.001
SBP (mmHg)	131.6 ± 16.2	122.3 ± 15.8	0.025	132.0 ± 16.9	0.901
DBP (mmHg)	82.9 ± 12.6	81.2 ± 9.8	0.531	82.3 ± 10.1	0.859
WC (cm)	85.4 ± 11.7	83.9 ± 12.5	0.110	82.9 ± 12.7	0.014
Neck circumference (cm)	35.1 ± 4.7	35.3 ± 4.4	0.568	34.2 ± 4.6	0.025
Physical activity (MET)	920 (60–2040)	1520 (420–3744)	0.173	840 (130–1060)	0.442
**Metabolic parameters**					
FPG (mg/dL)	102 ± 16.4	96.5 ± 12.1	0.051	100.8 ± 21.7	0.684
2 h-PG (mg/dL)	151.5 ± 49.7	142.9 ± 47.3	0.406	141.1 ± 52	0.377
HbA1c (%)	6.00 ± 0.25	6.03 ± 0.41^a^	0.676	5.89 ± 0.37	0.103
Matsuda Index	2.53(1.45–5.33)	3.91(2.17–7.77)	0.311^b^	7.49(1.42–11.85)	0.064^b^
HOMA-IR	3.72(1.35–10.11)	1.41(1.10–4.44)	0.010^b^	1.51(0.44–4.10)	0.036^b^
Insulinogenic Index	0.84(0.39–1.90)	0.51(0.15–0.79)	0.055^b^	0.52(0.33–1.74)	0.972^b^
Disposition Index	1.70(0.88–4.26)	1.25(0.70–1.92)	0.152^b^	2.22 (0.83–5.52)	0.311^b^
Total cholesterol (mg/dL)	208.4 ± 41.2	216.5 ± 48.7	0.420	208.8 ± 52.9	0.965
HDL cholesterol (mg/dL)	48.2 ± 11.4	52.5 ± 15.6	0.105	51.2 ± 17.1	0.258
LDL cholesterol (mg/dL)	130.5 ± 40.4	134.2 ± 44.6	0.682	134.0 ± 45.7	0.679
Triglycerides (mg/dL)	179.2 ± 145.6	166.1 ± 95.3	0.684	135.8 ± 60.9	0.274
**Sleep parameters**					
PSQI	4.31 ± 2.98	3.08 ± 1.85	0.151	NA	NA
Sleep efficiency (%)	83.34 ± 7.50	86.50 ± 5.73	0.048	NA	NA
Sleep duration (min)	351.86 ± 44.37	363.85 ± 53.01	0.244	NA	NA
SD sleep duration (min)	54.30 ± 23.62	47.99 ± 20.31	0.546	NA	NA
SD MST^c^	0:26 ± 0:13	0:34 ± 0:26	0.167	NA	NA
Quality of life					
EQ-5D-5L	0.977 ± 0.033	0.994 ± 0.014	0.143	0.945 ± 0.064	0.116
VAS	81.08 ± 11.98	91.15 ± 9.61	0.012	83.46 ± 8.26	0.417

Data was presented as mean ± SD or IQR; ^a^, n = 12; ^b^, Wilcoxon Signed Ranks Test; ^c^, time presented as 24-h clock time

*PLWH* people living with HIV, *BW* body weight, *BMI* body mass index, *NAFLD* nonalcoholic fatty liver disease, *ART* antiretroviral therapy, *NNRTI* non-nucleoside reverse transcriptase inhibitor, *PI* protease inhibitor, *SBP* systolic blood pressure, *DBP* diastolic blood pressure, *WC* waist circumference, *MET* Metabolic Equivalents, *FPG* fasting plasma glucose, *2 h-PG* 2-h plasma glucose, *HbA1c* hemoglobin A1c, *HDL* high-density lipoprotein, *LDL* low-density lipoprotein, *PSQI* Pittsburgh Sleep Quality Index, *SD* standard deviation, *MST* mid-sleep time, *EQ-5D-5L* the European Quality of Life Measure-5 Domain-5-Level, *VAS* visual analog scale

^a^n = 12

^b^Wilcoxon Signed Ranks Test

^c^Time presented as 24-h clock time

For sleep parameters, sleep efficiency (a marker of sleep quality) significantly increased at 6 months, whereas there were no changes in other parameters (Table 2).

Quality of life assessed by EQ-5D-5L did not change. However, quality of life as assessed by VAS significantly increased at 6 months.

### Relationship between changes in BMI, physical activity, sleep parameters and changes in metabolic parameters during ILI

We further investigated the association between changes in BMI, physical activity, sleep parameters and changes in metabolic parameters at 6 months (Table 3). A decrease in BMI correlated to an increase in the Matsuda index. An increase in physical activity was associated with a reduction in HbA1c and insulinogenic index. For changes in sleep parameters, an increase in sleep duration was associated with a reduction in HbA1c and insulinogenic index. However, the associations became non-significant after controlling for changes in BMI or physical activity. In addition, an increase in sleep variability (SD sleep duration and SD MST) was significantly associated with an increase in 2 h-PG. The association between ∆SD sleep duration and 2 h-PG remained significant after adjusting for changes in BMI (b = 0.603, 95% CI: 0.146, 1.059, *p* = 0.015) or physical activity (b = 0.774, 95% CI: 0.339, 1.209, *p* = 0.003), but the ∆SD MST and 2 h-PG became non-significant after adjustment.

**Table 3:** Correlation between changes in BMI, physical activity, sleep parameters and changes in metabolic parameters between baseline and 6 months in 13 PLWH with prediabetes

	∆FPG	∆2 h-PG	∆HbA1c^a^	∆Matsuda index	∆HOMA IR	∆insulinogenic index	∆disposition index
∆BMI	0.505	0.495	0.322	− 0.560*	0.258	0.264	− 0.214
∆MET	0.076	0.190	− 0.634*	-0.019	0.212	− 0.595*	− 0.424
∆Sleep efficiency	-0.287	− 0.283	− 0.231	0.005	− 0.346	− 0.176	− 0.088
∆Sleep duration	0.097	0.113	− 0.587^*^	-0.044	− 0.192	− 0.571^*^	− 0.214
∆SD Sleep duration	-0.168	0.790^**^	0.000	0.363	0.374	0.137	− 0.016
∆SD MST	0.127	0.597*	− 0.098	0.148	− 0.016	− 0.269	− 0.132

Bold indicated statistically significant results

*PLWH* people living with HIV, *FPG* fasting plasma glucose, *2 h-PG* 2-h plasma glucose, *HbA1c* hemoglobin A1c, *BMI* body mass index, *SD* standard deviation, *MST* mid-sleep time

^a^n = 12; *,p < 0.05; **,p < 0.01

This ILI program was well-accepted. Over 90% of participants were satisfied. The program attendance was 84.6–100%, suggesting that the program was feasible in this setting.

## Discussion

In this pilot study of 20 normoglycemic and 19 prediabetic PLWH, we did not find differences in sleep characteristics between the two groups. The pilot six-month ILI program for PLWH with prediabetes was effective in improving diabetes risks, including a reduction in BW, BMI, WC and HOMA-IR, which were maintained at 12 months. The program was feasible and acceptable, as evaluated by the participants’ attendance and satisfaction. Furthermore, the changes in sleep variability were a novel factor associated with changes in metabolic parameters after ILI. An increase in SD sleep duration was significantly associated with increased 2 h-PG regardless of the changes in BMI and physical activity. These results from the present study suggest that ILI is effective and feasible in reducing diabetes risks in PLWH with prediabetes, and that sleep modification could possibly be complimentary to ILI. Therefore, a larger dedicated RCT should be performed to confirm these findings.

Of note, the magnitude of BW reductions in the present study was smaller than the pre-specified goal of the program. However, this was comparable to the findings from a Community-Based DPP in Thailand [36]. This might be due to the lower baseline weight. Nevertheless, the weight reduction was accompanied by a significant reduction in HOMA-IR over the 12-month period.

This is the first study demonstrating that objectively measured sleep duration and sleep variability may play a role in the effectiveness of ILI in PLWH. However, the findings should be interpreted with caution due to the small number of subjects. Insufficient sleep demonstrated to increase diabetes risk in some studies [11, 37]. Furthermore, maintaining adequate sleep was also shown to be beneficial during a weight loss study [38]. In a study of ILI, individuals with prediabetes who reported ≤ 6 h/night of sleep at enrollment had a significantly higher rate of incident diabetes, as well as less weight loss [39], compared to those sleeping 7 h. Our results in PLWH are in agreement with this data, as we demonstrated that a decrease in objectively measured sleep duration was associated with an increase in HbA1c. In addition, this is the first study that demonstrated that increased sleep variability was associated with an increase in 2 h-PG after ILI. Increasing ten minutes of SD sleep duration increased 2 h-PG 6 mg/dL after adjusting for changes in BMI, and increased 2 h-PG 7.7 mg/dL after adjusting for changes in physical activity. Sleep variability has been increasingly recognized to be related to metabolic health. This could potentially be related to the shift in timing of sleep which could affect the body’s circadian regulation, and hence, affect metabolism. Sleep variability has been shown to be associated with glycemic control in people with type 1 diabetes [13, 14], less physical activity [40], higher prevalence and incidence of metabolic abnormalities [41], and an unhealthy diet [42]. Our pilot results suggested that maintaining adequate and regular sleep could potentially be beneficial in reducing diabetes risks during ILI in PLWH.

In conclusion, intensive lifestyle interventions in PLWH with prediabetes is feasible and effective in improving metabolic control. The preliminary results suggest that the effects of ILI are modulated by sleep duration and sleep variability. This highlights a possibility to apply the ILI program and sleep adjustment as a diabetic prevention program in this high-risk group for T2DM.

## Limitation

We recruited only PLWH in this study, therefore, we lacked a comparison of the differences in sleep variability and glucose metabolism in individuals without HIV. In this study, participants in normoglycemia and prediabetes groups were not matched, however, the demographic and anthropometric parameters were similar. In addition, we recruited PLWH with prediabetes who received ART with complete viral suppression. Thus, our results may not be applicable to PLWH without successful ART. Since the cultural and socioeconomic needs vary between different ethnic groups, the results may not be implied for non-Asian populations. The number of study participants was small. Lastly, more detailed objective sleep and metabolic measurements such as body fat composition, hyperinsulinemic euglycemic clamp and OSA assessment were not performed in this study.”

## Data Availability

The datasets used and/or analyzed during this study are available from the corresponding author on reasonable request.

## Abbreviations

2 h-PG: 2-H plasma glucose
ART: Antiretroviral therapy
BMI: Body mass index
DPP: Diabetes Prevention Program
EQ-5D-5L: The European Quality of Life Measure-5 Domain-5-Level
FPG: Fasting plasma glucose
GPAQ: Global Physical Activity Questionnaire
h: Hour
HbA1c: Hemoglobin A1c
HIV: Human Immunodeficiency Virus
ILI: Intensive lifestyle interventions
METs: Metabolic Equivalents
MST: Mid-sleep time
OGTT: Oral glucose tolerance test
PSQI: Pittsburgh Sleep Quality Index
RCT: Randomized controlled trial
SDs: Standard deviations
T2DM: Type 2 diabetes mellitus
VAS: Visual analog scale
WC: Waist circumference
